# Catheter Ablation for Atrial Fibrillation in Patients with Heart Failure: Current Evidence and Future Opportunities

**DOI:** 10.3390/jpm13091394

**Published:** 2023-09-18

**Authors:** Sho Suzuki, Takeshi Kitai, John Skoularigis, Kyriakos Spiliopoulos, Andrew Xanthopoulos

**Affiliations:** 1 Department of Cardiovascular Medicine, National Cerebral and Cardiovascular Center, Osaka 564-8565, Japan; 2 Department of Cardiovascular Medicine, Shinshu University School of Medicine, Nagano 390-8621, Japan; 3 Department of Cardiology, University Hospital of Larissa, 41110 Larissa, Greece; 4 Department of Cardiothoracic Surgery, University of Thessaly, Biopolis, 41110 Larissa, Greece

**Keywords:** atrial fibrillation, heart failure, catheter ablation, review

## Abstract

Atrial fibrillation (AF) and heart failure (HF) are highly prevalent cardiac disorders worldwide, and both are associated with poor prognosis. The incidence of AF and HF has been increasing substantially in recent years, mainly due to the progressive aging of the population. These disorders often coexist, and may have a causal relationship, with one contributing to the development or progression of the other. AF is a significant risk factor for adverse outcomes in HF patients, including mortality, hospitalization, and stroke. Although the optimal treatment for AF with HF remains unclear, catheter ablation (CA) has emerged as a promising treatment option. This review provides a comprehensive overview of the current scientific evidence regarding the efficacy of CA for managing AF in HF patients. In addition, the potential benefits and risks associated with CA are also discussed. We will also explore the factors that may influence treatment outcomes and highlight the remaining gaps in knowledge in this field.

## 1. Introduction

Atrial fibrillation (AF) and heart failure (HF) are both common cardiac disorders associated with poor prognosis [1,2,3,4]. These conditions often coexist, and one potentially leads to the other [5]. In patients with HF, AF is present in 10 to 60% of cases [1,5,6,7,8] and is more common in HF with preserved ejection fraction (HFpEF; left ventricular ejection fraction (LVEF) ≥ 50%) than in HF with mildly reduced ejection fraction (HFmrEF; LVEF 41–49%) or HF with reduced ejection fraction (HFrEF; LVEF ≤ 40%) [7,9]. The coexistence of AF and HF is associated with increased risks of mortality, HF hospitalization, and stroke [5,6,7,9]. The absence of an atrial kick and the irregular rhythm of the ventricle in AF will decrease stroke volume and make HF more difficult to control. In addition, increased heart rate in AF will shorten the filling time, which may lead to further deterioration of cardiac function [10]. Conversely, myocardial remodeling and activation of neurohormonal systems in HF risk the development of AF [11,12].

Over the past three decades, the prognosis of HFrEF has improved due to stepwise advancements in pharmacological/non-pharmacological treatment [13,14]. However, AF is still an independent risk factor for poor prognosis in these patients [5,7,9], and radical treatment should be considered.

The current guidelines recommend catheter ablation (CA) in patients with AF and HF who remain symptomatic on medical therapy, with an emphasis on HFrEF while acknowledging the importance of patient selection [15,16,17,18]. Over the past decade, advances in CA technology have resulted in improved success rates and a reduced risk of complications, making CA a viable option for many patients with AF and HF. In this review, we provide a comprehensive overview of the current scientific evidence focusing on CA against AF patients with HF and discuss the inconsistent treatment effect in the literature.

## 2. Catheter Ablation in Patients with Atrial Fibrillation and Heart Failure with Reduced Ejection Fraction

The optimal role of CA in managing AF patients with HFrEF is currently less clearly defined than in AF patients without HF. While current guidelines recommend CA for rhythm control in symptomatic, recurrent, and drug-resistant AF patients without HF, its use in those with HFrEF remains a topic of ongoing debate among researchers and clinicians [15,19]. In AF patients with HF, CA is often considered the second-line therapy following medical treatment, except in particular conditions, such as when tachycardia-induced cardiomyopathy is highly suspected [15,16,17,18]. However, in AF patients with HF, it is difficult to determine whether their symptoms are arrhythmia-related, and CA should be considered if HF is unmanageable regardless of symptoms. Especially in HF, the negative inotropic effect of anti-arrhythmic drugs (AADs) may be a risk for worsening HF, and an earlier CA may be worth considering. Current guidelines state that CA for AF with HF improves quality of life (QOL), symptoms, and LVEF, while only mentioning the potential effect of improving prognosis [15,16,17,18].

To date, a total of nine prospective randomized controlled trials (RCTs) have been reported, investigating the efficacy of CA in patients with AF and HFrEF, with some trials also including HFmrEF and HFpEF (Table 1) [20,21,22,23,24,25,26,27,28]. In patients with symptomatic HF (New York Heart Association (NYHA) class ≥ II), the effect of CA was compared with medical rate/rhythm control or atrioventricular-node ablation with biventricular pacing. Here, we summarize the study results of the completed RCTs.

### 2.1. Effect on Left Ventricular Ejection Fraction in HFrEF

**Figure 1:**
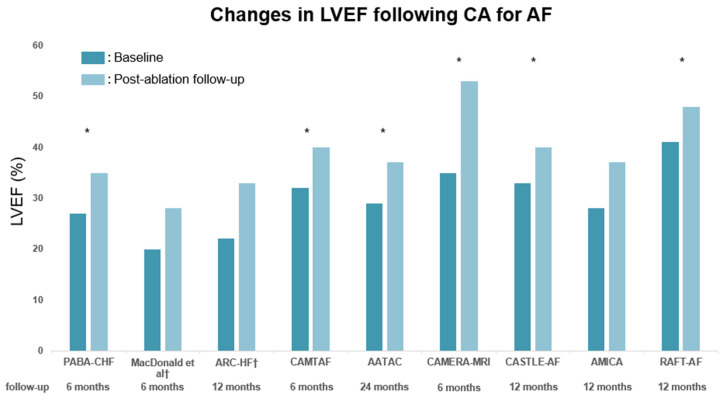
Change in left ventricular ejection fraction (LVEF) from baseline to post-ablation follow-up in published RCTs. The studies in which LVEF was measured by radionuclide ventriculography are indicated by daggers. Asterisks indicate trials that demonstrated statistically significant differences compared with the control group. The dark-blue line represents the baseline LVEF; the light-blue line represents the LVEF at post-ablation follow-up. LVEF, left ventricular ejection fraction [20,21,22,23,24,25,26,27,28].

All nine trials assessed the effect of CA on LVEF, five of which investigated the increase in LVEF as the trial’s primary endpoint. Seven trials evaluated LVEF with transthoracic echocardiography (TTE), and the remaining two evaluated LVEF with radionuclide ventriculography. Notably, a rhythm control with CA improved LVEF compared to baseline in all studies (Figure 1). Six studies showed a significant increase in LVEF in the CA group compared with pharmacological treatment (rate/rhythm control) or atrioventricular-node ablation with biventricular pacing. In contrast, three studies did not show significant differences in LVEF improvement between CA and pharmacological treatment. MacDonald et al. enrolled 41 patients with persistent AF and symptomatic HF with LVEF < 35% (mean 18%) measured by radionuclide ventriculography, and compared the change in LVEF measured by cardiac magnetic resonance after six months between CA and pharmacological rate control [21]. The increase in cardiac magnetic resonance LVEF in the CA group was +5% compared with +3% in the pharmacological treatment group (*p* = 0.6). However, in this study, the sinus rhythm (SR) restoration rate in the CA group was only 50%, and half of the patients undergoing CA were in AF after six months. The 10 patients who were in SR after six months had a significantly higher increase in LVEF of 10% compared to 1.5% who remained AF (*p* = 0.008). As the authors pointed out, the low success rate of CA would have affected the results. The ARC-HF (A Randomised Trial to Assess Catheter Ablation Versus Rate Control in the Management of Persistent Atrial Fibrillation in Chronic Heart Failure) trial [22], compared the effect of CA with pharmacological rate control in 52 patients with persistent AF and symptomatic HF with LVEF <35% (mean 24%) measured by radionuclide ventriculography. There was a nonsignificant trend toward LVEF improvement (+10.9% vs. +5.4%, *p* = 0.055) in the CA arm. The recently published AMICA (Atrial Fibrillation Management in Congestive Heart Failure With Ablation) trial [27] compared the effect of CA with best medical therapy (rate/rhythm control) in 140 patients with persistent AF and symptomatic HF with LVEF ≤35% (mean 26%) measured by TTE. There was no difference in LVEF improvement (+9% vs. +7%, *p* = 0.36) under a 74% maintenance of SR in patients who underwent CA. These study results differed from other previous RCTs, including results from the AATAC (Ablation vs. Amiodarone for Treatment of Atrial Fibrillation in Patients With Congestive Heart Failure and an Implanted ICD/CRTD) trial [24] and the CASTLE-AF (Catheter Ablation versus Standard Conventional Therapy in Patients with Left Ventricular Dysfunction and Atrial Fibrillation) trial [26]. One possible reason for the inconsistent results is that the AMICA trial also showed a 7.3% increase in LVEF in the medical therapy group; the LVEF improvement in the medical therapy group was higher than in other studies, which may have contributed to the study results. The LVEF improvement in the CA group in the AMICA trial was comparable to other studies. Another reason may be that the three studies [21,22,27] which did not show positive results in LVEF improvement had lower basal LVEF than other studies. As the CAMERA-MRI (Catheter Ablation Versus Medical Rate Control in Atrial Fibrillation and Systolic Dysfunction) trial [25] reported that a greater improvement in LVEF was observed in patients without late gadolinium enhancement, there may be a less beneficial effect in LVEF improvement with CA in severely impaired cardiac function. In addition, two of the nine trials included paroxysmal AF in their study, while the other seven consisted of patients with persistent AF. Although the mean left atrium diameter was 46–51 mm, with no apparent correlation with paroxysmal AF, the mean duration of AF varied, ranging from 0.7 to 4.4 years. While not immediately evident, this could suggest potential discrepancies in the stage of AF represented across the trials. Identifying a precise LVEF threshold to delineate the efficacy of CA would be clinically beneficial. However, to date, empirical evidence supporting such a threshold remains elusive, necessitating further rigorous studies in the future. In any case, the improvement of LVEF with CA in patients with AF and HFrEF may have been somewhat established, especially in earlier treatment.

### 2.2. Effect on Quality of Life in HFrEF

**Figure 2:**
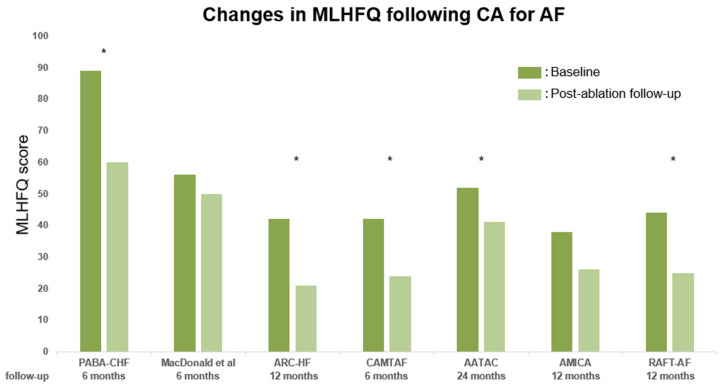
Changes in Minnesota Living with Heart Failure questionnaire (MLHFQ) scores from baseline to post-ablation follow-up in published RCTs. Asterisks indicate trials that demonstrated statistically significant differences compared with the control group. The dark-green line represents the baseline LVEF; the light-green line represents the LVEF at post-ablation follow-up. MLHFQ, Minnesota Living with Heart Failure questionnaire [20,21,22,23,24,27,28].

Seven trials [20,21,22,23,24,27,28] investigated the impact of CA on QOL by evaluating the changes in Minnesota Living with Heart Failure questionnaire (MLHFQ) score (Figure 2). The MLHFQ is one of the most widely used questionnaires to assess health-related QOL for patients with HF, which has demonstrated good psychometric properties in numerous studies [29,30]. The questionnaire comprises 21 questions about physical, emotional, and socioeconomic situations. Patients mark a 0–5 scale to each question, and the summation of all 21 questions will produce the MLHFQ score. A lower MLHFQ score is indicative of a better QOL for the patient. Patients’ QOL improved after a rhythm control with CA in all studies. Five studies [20,22,23,24,28] showed a significant decrease in MLHFQ scores in the CA group compared with pharmacological treatment (rate/rhythm control) or atrioventricular-node ablation with biventricular pacing. The two studies which did not show a significant difference in MLHFQ score improvement between CA and pharmacological treatment were the RCT reported from Scotland [21], and the AMICA trial [27]. It is plausible that the negative results in these two studies can be attributed to the same factors as those affecting LVEF. Therefore, it is possible that the effectiveness of CA in improving QOL was not adequately demonstrated in these studies.

### 2.3. Effect on Functional Capacity in HFrEF

**Figure 3:**
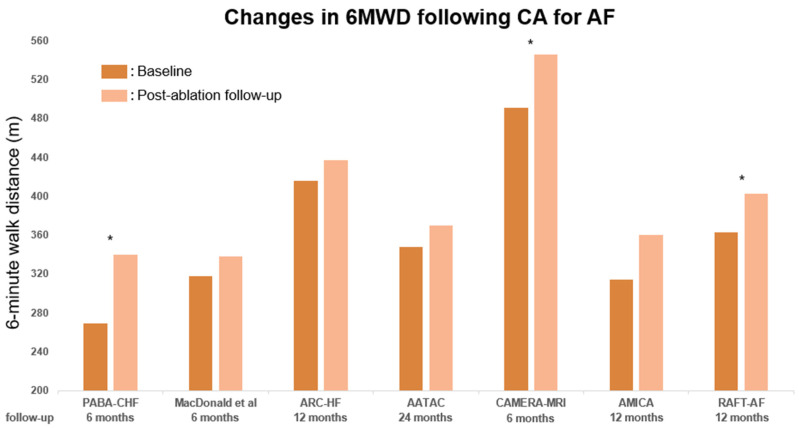
Change in 6 min walk distance (6MWD) from baseline to post-ablation follow-up in published RCTs. Asterisks indicate trials that demonstrated statistically significant differences compared with the control group. The dark-orange line represents the baseline LVEF; the light-orange line represents the LVEF at post-ablation follow-up. 6MWD, six-minute walk distance [20,21,22,23,24,27,28].

All of the nine trials examined the impact of CA on functional capacity, with eight of them focusing on the change in the six-minute walk distance (6MWD) [20,21,22,24,25,26,27,28], while two studies investigated the change in peak oxygen consumption, with one of them examining both 6MWD and peak oxygen consumption [22,23]. The two studies that assessed the peak oxygen consumption change observed significant increases in the ablation arm compared to the pharmacological treatment arm. Regarding 6MWD, all studies showed an increase after a rhythm control with CA (Figure 3). While three studies [20,24,28] observed a significant improvement in 6MWD in the CA arm, four studies reported no significant increase in walking distance compared to the pharmacological treatment (rate/rhythm control) or atrioventricular-node ablation with biventricular pacing. Interestingly, in the CASTLE-AF trial [26], the difference in 6MWD improvement between the CA group and the pharmacological treatment group was significant at 12 months’ follow-up (+41 m vs. +1 m, *p* = 0.001, n = 294) but not at 36 months’ follow-up (+10.5 m vs. +20 m, *p* = 0.38, n = 165) and 60 months’ follow-up (+0 m vs. −30 m, *p* = 0.67, n = 85). In addition, the distance in both groups varied through the follow-up period (observing a better increase in the pharmacological treatment arm at 36 months, but turning to a decrease after 60 months). It should be noted that due to the progressive nature of HFrEF and the recurrent nature of AF over time, accurately assessing the precise effect of CA on functional capacity through 6MWD may be challenging. Nonetheless, as demonstrated in Figure 3, it is apparent that CA confers at least a transient improvement in functional capacity.

### 2.4. Prognostic Impact of Catheter Ablation in HFrEF

**Figure 4:**
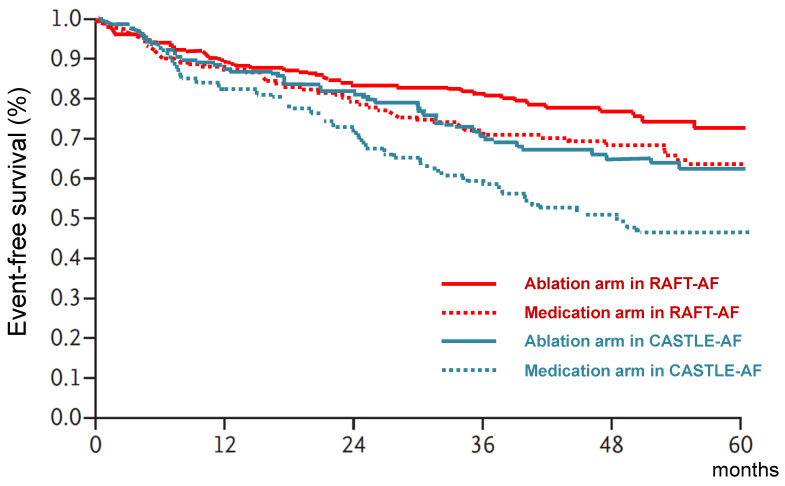
Combined Kaplan–Meier plots of the primary outcome in the CASTLE-AF and RAFT-AF trials.

The prognostic impact of CA in patients with HFrEF was evaluated by three trials [24,26,28]. The CASTLE-AF trial [26] was the first prospective multicenter clinical trial to evaluate the long-term prognostic impact of CA in patients with AF and HFrEF. This study compared CA with pharmacological treatment (rate/rhythm control) in 363 patients with symptomatic AF (including 118 patients with paroxysmal AF) and HF with LVEF < 35% (mean 32%). The primary endpoint was a composite of death from any cause or worsening of HF that led to unplanned overnight hospitalization. During a mean follow-up period of 37.6 ± 20.4 months, the composite primary endpoint occurred in significantly fewer patients in the CA group than in the pharmacological treatment group (51/179 patients (28.5%) vs. 82/184 patients (44.6%), *p* = 0.006). In the secondary endpoints, the individual risk of all-cause death, cardiovascular death, and HF-related admission was also lower in the CA group. On the other hand, the recently published RAFT-AF (Rhythm Control—Catheter Ablation With or Without Anti-arrhythmic Drug Control of Maintaining Sinus Rhythm Versus Rate Control With Medical Therapy and/or Atrio-ventricular Junction Ablation and Pacemaker Treatment for Atrial Fibrillation) trial [28] showed inconsistent results with the CASTLE-AF trial. The RAFT-AF trial compared the effect of CA with pharmacological rate control and/or atrioventricular-node ablation with biventricular pacing in 411 patients with symptomatic AF (including 30 patients with paroxysmal AF) and HF (mean LVEF 41%). The primary endpoint was a composite of death from any cause or HF events defined as an admission to a healthcare facility for >24 h or clinically worsening HF leading to the administration of intravenous in an emergency department or unscheduled visit to a healthcare provider, an increase in chronic HF therapy. During a median follow-up period of 37.4 [interquartile range 24.7–53.7] months, the composite primary endpoint occurred in 50/214 (23.4%) in the CA group compared with 64/197 (32.5%) in the rate-control group (*p* = 0.066). The combined Kaplan–Meier plots of each primary outcome in the two trials are shown in Figure 4. Two possible reasons for the inconsistent results of the two studies are as follows. First, in the RAFT-AF trial, atrioventricular-node ablation with biventricular pacing was performed in patients who were uncontrollable with pharmacological rate control, and rate control was more rigorous than in the CASTLE-AF trial. This could be one of the reasons why the RAFT-AF trial had a lower event rate in the control group than in the CASTLE-AF trial, which may have influenced the results. Second, there were no limits of LVEF in the inclusion criteria of the RAFT-AF trial, and patients with HFmrEF and HFpEF were included in the study. As noted in the later section, the effect of CA in patients with HFmrEF and HFpEF are still not well established, and further research is necessary. Moreover, sub-group analysis in the RAFT-AF trial showed that patients with higher LVEF contributed more to the negative results (patients with LVEF ≤45% showed more favorable results for CA than those with LVEF > 45%). The mean LVEF of 41% in the RAFT-AF trial was the highest in all nine trials, which could be another reason for the discrepancy in the Kaplan–Meier curve between the two studies. The AATAC trial [24] investigated the effect of CA in 203 patients with persistent AF and symptomatic HF with LVEF < 40% (mean 30%) and compared the risk of all-cause mortality and HF-related unplanned hospitalization with amiodarone administration. Over a 2-year follow-up, the risk of death and HF-related unplanned hospitalization were both significantly lower in the CA arm. Based on the available evidence, it appears that CA may have a positive impact on the prognosis of patients with AF and HFrEF. However, there is a need for further studies to confirm these findings.

### 2.5. Complication Risks and Atrial Fibrillation Recurrence

Data from prospective registry studies indicate that complications occur in around 4–14% of patients who undergo CA for AF, with 2–3% of these complications being potentially life-threatening [15,31,32,33,34,35,36,37,38,39,40,41,42]. The complication rates were 0–15% in the nine RCTs [20,21,22,23,24,25,26,27,28] regarding CA for AF patients with HF, 0–4.7% of which were potentially life-threatening, and the frequency of complications was similar (exceptionally, MacDonald et al. reported fatal complications in 3 of 27 patients). However, patients who underwent CA in the RCTs of AF and HF were relatively young, around a mean age of 60. Older patients will be eligible in actual clinical practice, which needs to be noted.

Given the added consideration of repeated CA procedures and the use of AADs, determining the exact recurrence rate and the follow-up period for AF recurrence presents a challenge. AF recurrence is monitored through a 12-lead electrocardiogram, 24/48 h Holter monitoring, implantable loop recorders, or implantable cardioverter defibrillator (ICD)/cardiac resynchronization therapy defibrillator (CRTD). The general SR maintenance rates after CA have been reported to range from 63 to 84% in most studies with a follow-up period of 2 to 5 years [43,44,45,46,47,48,49,50]. Although SR maintenance rates in RCTs similarly range from 63 to 86%, the follow-up was relatively shorter, ranging from 0.5 to 2 years [20,22,23,24,25,26,27,28]. The CASTLE-AF trial was the only study that investigated the long-term (5 years) effect of CA in patients with AF and HFrEF. This may be explained by the natural progression of HFrEF, which generally has a poor prognosis compared to conditions without HF.

Several risk factors for AF recurrence after AF ablation have been identified, including patient age, left atrium size, AF duration, renal dysfunction, and LGE in MRI [15,51,52,53,54,55,56,57]. In particular, left atrium volume and left atrium structure have been reported to not only be significant predictors of AF recurrence after CA [55,58,59] but also be associated with left ventricular function [60,61]. These are essential factors to consider in treatment strategies for both AF and HF. It is evident that there is a lack of consensus among the various risk prediction models currently available to identify patients who are at a higher risk of AF recurrence following CA [62,63,64,65,66,67,68,69,70]. Hence, additional investigations are required to identify risk factors for AF recurrence in patients with HF, which would assist in determining patient selection for CA.

### 2.6. Catheter Ablation for Advanced Heart Failure

Although CA has demonstrated beneficial effects in several studies and should be considered a first-line treatment option for selected patients with HFrEF, its efficacy in the context of advanced or end-stage HF remains to be fully elucidated. This is primarily due to the exclusion of patients with advanced HF from these studies.

Recently, the CASTLE-HTx (Catheter Ablation for Atrial Fibrillation in Patients with End-Stage Heart Failure and Eligibility for Heart Transplantation) trial [71] assessed the safety and efficacy of CA in patients with end-stage HF who were considered for heart transplantation or implantation of a left ventricular assist device. This study compared CA with pharmacological treatment in 194 patients with symptomatic AF, with 68% of the enrolled patients classified as NYHA class ≥ III. The primary endpoint was a composite of death from any cause, implantation of a left ventricular assist device, or urgent heart transplantation. During a median follow-up period of 18.0 [14.6–22.6] months, the composite primary endpoint occurred in significantly fewer patients in the CA group compared to the pharmacological treatment group (8/97 patients (8.2%) vs. 29/97 patients (30.0%), *p* < 0.001). These findings suggest that CA might also hold promise as an effective therapeutic strategy in treating advanced HF.

## 3. Atrial Fibrillation and Heart Failure with Preserved Ejection Fraction

AF and HFpEF are age-related, frequently coexisting, and likewise share common mechanisms and risk factors [7,72,73,74,75]. Previous studies clearly show that AF is associated with poor prognosis in patients with HFpEF [75,76,77,78,79,80]. Thus, early treatment of AF is equally important in patients with HFpEF. Atrial remodeling in HFpEF typically results in impaired ventricular diastolic function [61], and the maintenance of SR would be key to improve clinical outcomes in these patients. However, limited data are available regarding the effectiveness, safety, and rate of maintaining sinus rhythm with CA in patients diagnosed with both AF and HFpEF. Observational studies investigating the prognostic significance of CA compared with pharmacological therapy are shown in Table 2. Among the three studies, two single-center retrospective studies reported that CA was independently associated with better prognosis in a small population [81,82]. Although a study from the United States national database showed no association between CA and better prognosis, this study lacked data regarding LVEF and the definition of HFpEF [83]. A sub-analysis from the CABANA trial [84], which investigated the prognostic impact of CA in AF patients with HF (whose median LVEF was 55%), showed a significant reduction of the primary composite outcome (death, disabling stroke, serious bleeding, and cardiac arrest) in the CA arm [85]. The efficiency of CA in cases where AF leads to HFpEF and vice versa warrants future investigation. Currently, there are no completed RCTs that investigate the prognostic impact of CA compared with pharmacological therapy in patients with AF and HFpEF. It is reasonable to await the results of the upcoming AMPERE (Ablation Versus Medical Management of Atrial Fibrillation in HFpEF) trial and the CABA-HFPEF (CAtheter-Based Ablation of Atrial Fibrillation Compared to Conventional Treatment in Patients With Heart Failure With Preserved Ejection Fraction) trial for further evidence.

## 4. Conclusions and Future Perspectives

This review has synthesized the available literature on the utilization of CA as a potential treatment for patients suffering from both AF and HF. While CA has emerged as a promising therapeutic option for this population, several unresolved issues persist that need to be addressed to optimize its application.

One of the primary challenges associated with CA for AF in HF patients is the controversial prognostic impact of the treatment. There are several factors that may influence the effect of CA in HF patients including the duration of AF, the progression of underlying cardiac disease, and the severity of HF. These factors must be considered when evaluating the effectiveness of CA and require further investigation. Another important consideration is the need to establish patient selection criteria and predictors of AF recurrence in AF patients with HF. This will require further research to determine which patients are most likely to benefit from CA and what the best approach is to predict the risk of AF recurrence. Finally, the effect of CA in AF patients with HFpEF remains unclear, and further research is needed to understand the potential benefits and risks associated with the treatment in this population.

## Figures and Tables

**Table 1:** Randomized controlled trials of catheter ablation in patients with atrial fibrillation and heart failure with reduced ejection fraction.

Trials (RCTs)	Sample Size	Patient Population	Comparison Group	Primary Outcome	Follow-Up (Years)	LVEF (Mean)	Findings
(publication year) Ref	(number of PAF)						
**PABA-CHF** (2008) [20]	81 (42)	Symptomatic AF, NYHA II–III,	AV node ablation + BiV pacing	Composite of LVEF (TTE),	0.5	28	Beneficial effect of CA on
		and LVEF ≤ 40% (TTE)		6MWD, and MLWHQ score			LVEF, 6MWD, QOL
**MacDonald et al.** (2011) [21]	41 (0)	Persistent AF, NYHA II–IV,	Pharmacological rate control	Change in LVEF (CMR)	0.5	18	No beneficial effect of CA on
		and LVEF < 35% (RNVG)				(RNVG)	LVEF (CMR), 6MWD, QOL
							AF recurrence 50% in CA arm
**ARC-HF** (2013) [22]	52 (0)	Persistent AF, NYHA II–IV,	Pharmacological rate control	Change in peak VO_2_	1	24	Beneficial effect of CA on
		and LVEF ≤ 35% (RNVG)					peak VO_2_, QOL
**CAMTAF** (2014) [23]	50 (0)	Persistent AF, NYHA II–IV,	Pharmacological rate control	Change in LVEF (TTE)	0.5	32	Beneficial effect of CA on
		and LVEF < 50% (TTE)					LVEF, peak VO_2_, QOL
**AATAC** (2016) [24]	203 (0)	Persistent AF, NYHA II–IV,	Amiodarone	Recurrence of AF	2	30	Beneficial effect of CA on
		LVEF < 40% (TTE), and ICD/CRTD					LVEF, 6MWD, QOL, and
							prognosis (death + HF)
**CAMERA-MRI** (2017) [25]	68 (0)	Persistent AF, idiopathic cardiomyopathy	Pharmacological rate control	Change in LVEF (CMR)	0.5	33	Beneficial effect of CA on
		NYHA II–IV, and LVEF ≤ 45% (CMR)					LVEF (especially in patients
							with non-LGE in CMR)
**CASTLE-AF** (2018) [26]	363 (118)	Symptomatic AF, NYHA II–IV,	Pharmacological treatment	Composite of death and	5	32	Beneficial effect of CA on
		LVEF < 35% (TTE), and ICD/CRTD	(rate/rhythm control)	HF hospitalization	(median 3.2)		prognosis (death + HF),
							and LVEF
**AMICA** (2019) [27]	140 (0)	Persistent AF, NYHA II–III,	Pharmacological treatment	Change in LVEF (TTE)	1	26	No beneficial effect of CA on
		LVEF < 35% (TTE), and ICD/CRTD	(rate/rhythm control)				LVEF, 6MWD, QOL
**RAFT-AF** (2022) [28]	411 (30)	Symptomatic AF, NYHA II–III,	Pharmacological rate control	Composite of death and	2	41	Nonsignificant trend for improved
		HF, elevated NT-proBNP	/AV node ablation + BiV pacing	HF events			prognosis (death + HF) with CA
							Beneficial effect on LVEF,
							6MWD, QOL

Abbreviations: AF = atrial fibrillation; AV = atrioventricular; BiV = biventricular; CA = catheter ablation; CMR = cardiovascular magnetic resonance; CRTD = cardiac resynchronization therapy defibrillator; HF = heart failure; ICD = implantable cardioverter defibrillator; LGE = late gadolinium enhancement; LVEF = left ventricular ejection fraction; NT-proBNP = N-terminal pro brain natriuretic peptide; NYHA = New York Heart Association; PAF = paroxysmal atrial fibrillation; QOL = quality of life; RCT = randomized controlled trial; RNVG = radionuclide ventriculography; TTE = transthoracic echocardiography; VO_2_ = oxygen consumption; 6MWD = six-minute walk distance.

**Table 2:** Observational studies comparing catheter ablation and pharmacological therapy in patients with atrial fibrillation and heart failure with preserved ejection fraction.

Studies	Study Design	Sample Size	Primary Outcome	Follow-Up (Years)	Findings
(publication year) Ref					
**Fukui et al.**	Single-center retrospective	85	HF readmission	2.2	Significant association between CA
(2020) [81]	cohort study				and a lower risk of HF readmission
**Arora et al.**	Retrospective cohort study	56,395	Death + HF readmission	1	No association between CA and
(2020) [83]	using a national database				better prognosis
**Rattka et al.**	Single-center retrospective	127	Death + HF readmission	1.5	Significant association between CA
(2021) [82]	cohort study				and a lower risk of the primary outcome

Abbreviations: CA = catheter ablation; HF = heart failure.

## Data Availability

Not applicable.
